# Effectiveness of an individually tailored home-based exercise rogramme for pre-frail older adults, driven by a tablet application and mobility monitoring: a pilot study

**DOI:** 10.1186/s11556-021-00264-y

**Published:** 2021-06-21

**Authors:** Hilde A. E. Geraedts, Hidde Dijkstra, Wei Zhang, Francisco Ibarra, Iman Khaghani Far, Wiebren Zijlstra, Martin Stevens

**Affiliations:** 1 Center for Human Movement Sciences, University of Groningen, University Medical Center Groningen, Groningen, The Netherlands; 2 Department of Orthopaedics, University of Groningen, University Medical Center Groningen, P.O. BOX 30.001, 9700 RB Groningen, The Netherlands; 3 Philips Research Europe, Eindhoven, The Netherlands; 4 Department of Information Engineering and Computer Science, University of Trento, Trento, TN Italy; 5 Department of Electronics and Informatics, “Nuestra Señora de la Asunción” Catholic University, Asunción, Paraguay; 6 Institute of Movement and Sport Gerontology, German Sport University Cologne, Cologne, Germany

**Keywords:** Body-worn sensors, Home-based exercise programs, Remote physical activity monitoring, Older adults, Daily physical activity

## Abstract

**Objectives:**

To gain first insight into the effectiveness of a home-based exercise programme for pre-frail older adults with independent use of novel ICT technology.

**Methods:**

A pilot study. Forty pre-frail older adults joined a six-month home-based exercise programme using a tablet PC for exercise administration and feedback, and a necklace-worn motion sensor for daily physical activity registration. Participants received weekly telephone supervision during the first 3 months and exercised independently without supervision from a coach during the last 3 months. Functional performance and daily physical activity were assessed at baseline, after three and 6 months.

**Results:**

Twenty-one participants completed the programme. Overall, functional performance showed positive results varying from (very) small to large effects (Cohen’s *d* 0.04–0.81), mainly during the supervised part of the intervention. Regarding daily physical activity, a slight improvement with (very) small effects (Cohen’s *d* 0.07–0.38), was observed for both self-reported and objectively measured physical activity during the supervised period. However, during the unsupervised period this pattern only continued for self-reported physical activity.

**Conclusion:**

This pilot study showed positive results varying from (very) small to large effects in levels and maintenance of functional performance and daily physical activity, especially during the supervised first 3 months.

Remote supervision seems to importantly affect effectiveness of a home-based exercise programme. Effectiveness of the programme and the exact contribution of its components should be further quantified in a randomized controlled trial.

**Practice implications:**

Home-based exercising using novel technology may be promising for functional performance and physical activity improvement in (pre-frail) older adults.

**Trial registration:**

Netherlands Trial Register (NTR); trial number NL4049. The study was prospectively registered (registration date 14/11/2013).

**Supplementary Information:**

The online version contains supplementary material available at 10.1186/s11556-021-00264-y.

## Introduction

Our ageing society and its associated higher demand on healthcare resources has created a growing need for programmes to prevent physical decline and preserve the health and independent functioning of older adults [1, 2]. A chronic condition of particular interest to ageing research and prevention of physical decline is frailty. Frailty is defined as ‘the state of vulnerability to stressors that is independent of any specific disease or disability but that is common in older people and predisposes them to various adverse health outcomes’ [3]. By this definition, 14.5% of Dutch men and 20.7% of Dutch women aged 65 years or older are frail [4], as are 7 to 12% of American older adults [5]. Frailty is considered a major predisposition for falls and chronic health conditions that hinder independent physical functioning [6].

Regular physical activity has been shown to preserve health and prevent frailty in ageing persons, thus facilitating longer independence with higher quality of life [7, 8]. Research shows that a mere 30 min a day of moderate physical activity contributes to preventing physical decline in pre-frail older adults [9]. It is therefore of great importance to stimulate exercising and physical activity in pre-frail older adults who are generally inactive [10]. Research indicates that home-based exercise programmes are promising towards improving the functional performance and health of pre-frail older adults. Previous studies found better scores on leg biceps strength, chair-rise time and gait velocity after 6 weeks of home-based exercises targeting strength, flexibility, balance, gait and cardiovascular fitness [11]. However, uptake and adherence among frail and pre-frail older adults is generally low for home-based exercise programmes for reasons such as fear of falling or injury, or the belief that being physically active is not for older adults [10, 12]. Low adherence can be considered a very important factor compromising the effectiveness of home-based programmes, therefore efforts need to be made to maximise adherence among pre-frail older persons.

Recent developments in technology such as wearable sensors and tablet use with mobile internet are promising towards providing home-based exercise programmes while preserving the benefits and adherence stimulators from supervised exercise training in an exercise facility or even group training [13]. For instance, small body-worn sensors integrating accelerometers and gyroscopes are being developed to unobtrusively measure physical activity in daily life [14–19]. The accurate objective physical activity data collected by these sensors can be used for motivational strategies enhancing daily physical activity behaviour [13, 20]. A combination of objective data collection from daily life, tablet-based exercise instructions and motivational strategies using these data seems to be an effective method to improve functional performance and daily physical activity [20]. Although one might not think of pre-frail older adults as the ideal target group for home-based exercise programmes using novel technology, research shows that tablet, computer and internet use among older adults is rising steeply [21–23]. Older adults could therefore benefit from these technologies in the near future. The effectiveness of using novel technology in home-based exercise programmes for pre-frail older adults to enhance daily physical activity requires further study though.

The aim of this study was therefore to gain initial insight into the effectiveness of a home-based exercise programme, based on the Otago Kitchen Table Exercise programme, for older adults with independent use of remote novel technology in a pilot set-up. Primary research question was: What is the potential effectiveness of the home-based exercise programme using novel technology for pre-frail older adults, as indicated by an improvement in their functional performance (leg muscle power, coordination and balance)? Secondary research question was: What is the potential effectiveness of the home-based exercise programme using novel technology for pre-frail older adults, as indicated by an increase in their daily physical activity?

## Methods

### Study design

This pilot study consisted of a six-month intervention. Subjects participated in a home-based exercise programme, with exercise instructions through videos on a tablet PC and daily physical activity registration through a necklace-worn sensor. During the supervised first 3 months, participants were contacted by phone for weekly coaching and help with the technology as needed. During the unsupervised last 3 months participants were not contacted by phone but could call their coach if they encountered problems. When issues couldn’t be solved by phone, the coach visited the participant at home. Both coaches involved in the exercise programme had a background in human movement sciences and/or physiotherapy, and were trained in motivational techniques for physical activity stimulation in older adults. Each participant was assigned to a coach. The study protocol was approved by the Medical Ethical Committee of University Medical Center Groningen (METc no. 2013/246). A full, detailed description of the study design is provided elsewhere [24].

### Subjects

Community-dwelling pre-frail older adults living in the northern Netherlands were recruited and informed consent was obtained. Inclusion criteria were being over age 70 and the ability to walk at least 10 m independently or using a walking aid. Subjects had to be pre-frail, as indicated by the Groningen Frailty Indicator (GFI) (score of 4 or 5 out of a range of 0–15), which denotes a minor elevated chance of loss of functionality and heightened disability [3, 25]. Subjects needed sufficient mental capacity to perform a tablet-assisted exercise programme independently at home, as indicated by the score on the GFI and an additional subjective check by the coach. Exclusion criteria were physical conditions that hamper safe independent execution of a home-based exercise programme or working with a tablet, such as Parkinson’s disease stage 4 or 5 or severe visual problems. Candidates were recruited between January and November 2014 by means of advertisements and leaflets for participants of the integrated care programme Embrace [26].

### Exercise programme

The exercise regimen consisted of lower-body strength and balance exercises based on the Otago Kitchen Table Exercise programme [27, 28]. Exercise included stepping out sideways, lifting the legs alternatingly to the buttock, rising on toes and heels, and lifting legs while seated. Exercise progressed in 18 levels, increasing the exercise burden by adding more repetitions and longer training time as well as incorporating the use of ankle weights in the higher levels. Level 1 consisted of a 10-min training and the exercise burden increased to 40 min/day in level 18. The exercise programme is shown in detail in an additional table in PDF format [see Additional file 1: “Additional file 1 Table exercise program contents.pdf]. Each level was presented with an instructional video on the tablet. All participants started at level 1 and could progress through the levels as desired. Participants exercised five times a week. Adherence to the program was calculated based on completion of exercise bouts. In addition to the strength and balance exercises, participants were encouraged to increase their daily overall physical activity by a visual graph showing their daily physical activity progression. The encouragement strategies employed were based upon the transtheoretical model of behaviour change (Stages of Change model) and social-cognitive theory. Both the exercise programme and the motivational strategies are explained in detail elsewhere [24].

### Technical applications

#### Sensor

The necklace-worn sensor package included a miniature hybrid sensor containing a 3D-MEMS accelerometer and a barometric pressure sensor. Accelerometry data were sampled at 50 Hz with a range of 8 g, barometric data were sampled at 25 Hz. A micro-SD card was used for storage and exchange of data. The sensor weighed about 30 g and measured 55x25x10mm (Research prototype, Philips Research, Eindhoven, The Netherlands). Participants were asked to connect the sensor to the tablet manually using a USB cable to transfer data and load the battery every night. The sensor was used to measure daily physical activity and the performance on the functional tests.

#### Tablet PC

**Fig. 1:**
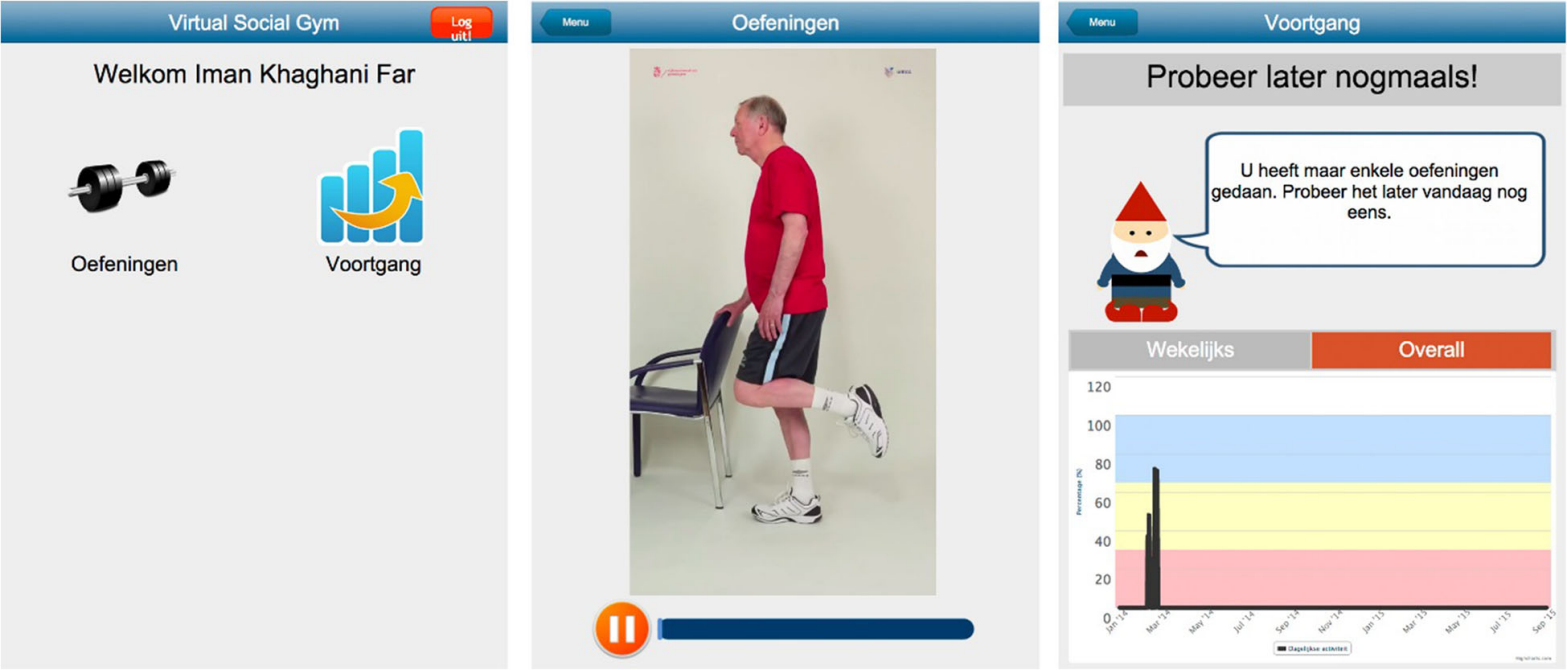
The web-based application

Participants received exercise instructions and distant feedback through a tablet PC, a Dell Latitude 10 with Windows 8 operating system. The tablet PC was adjusted to independent older adult use, keeping menus and necessary interaction as simple as possible. Exercise instructions were given using a web-based application (providing exercise videos and performance monitoring features; Fig. 1) that a participant could imitate. Participants could choose their own level of exercising in consultation with the coach. Each level had a different video showing the full exercise bout. The exercise programme was provided by an internet-based application running on a remote web server. Internet connection was provided by a 3G or 4G mobile internet card inserted into the tablet or by the participant’s own home Wi-Fi.

### Evaluation methods

#### Cohort characteristics

The following variables were collected at baseline: gender, age, body mass index, GFI score, Falls Efficacy Scale International (FES-I) score, computer experience, smartphone ownership and internet type.

#### Functional performance

Functional performance was assessed by means of three tests performed at a maximum but safe pace before the start of the intervention (baseline), after finishing the supervised first 3 months (post-test) and after finishing the unsupervised last 3 months (follow-up) at the participants’ homes. The tests were demonstrated and assisted by the researcher and the coach. First participants were asked to perform three separate sit-to-stand (STS) actions without using chair armrests [14, 18]. Based on the performance of the STS it was evaluated whether it was safe to ask the participant to continue the assessment with the two more difficult performance tests, Next, the Timed Up & Go test (TUG) was performed twice [29, 30]. The Five times Chair-Rise test (CR) was performed twice provided the participant could perform this task [17]. The tests were assessed based on data from the necklace-worn sensor as well as a stopwatch to measure time in seconds needed to perform the test [19]. The average time needed for repetitions of the functional performance tests per measurement point was calculated and used for comparison between the three measurement points. A drop in score means an improvement in functional performance. An extensive description of the measurement procedures has been published previously [24].

#### Daily physical activity

Daily physical activity was assessed by means of the self-reported Short QUestionnaire to ASses Health-enhancing physical activity (SQUASH) [31], which addresses questions about habitual physical activity level during a week and the necklace-worn sensor, which was worn daily throughout the entire intervention. As the sensor was a research prototype; the SQUASH was chosen to explain the research question regarding daily physical activity.

Sensor-wearing compliance was assessed by unplugging the sensor from the tablet – the sensor only recorded data when it was unplugged. These recorded data were uploaded into the system and automatically analysed at the next plug-in for sensor recharging. The coach could look into these data in the system daily, and when there were several consequent days of errant behaviour (flatline; only a small number of hours of data collected instead of at least 4 h; data that could not be translated by the sensor due to unclear patterns) the coach got in contact with the participant to check sensor-wearing adherence, progress and technology performance. Participants wore the sensor during the week before starting the exercise programme for baseline assessment. The week before the end of the supervised period was regarded as the post-test measurement, and the week before the end of the unsupervised period was regarded as the follow-up measurement of daily physical activity. Daily physical activity was expressed by means of Time-on-legs (TOL), defined as the percentage of time during the day spent active on one’s legs (standing, walking, shuffling and analogous activities) [19, 20]. The assessment weeks at the three measurement points provided seven measurement days each. Average physical activity over these 7 days was then calculated for all three measurement points. If one or more of the seven adjacent measurement days were not available, the physical activity measurement of an adjacent day prior to or after the seven-day measurement period was added to complete a seven-day measurement period. Physical activity as measured by the necklace-worn sensor was compared between the three measurement points.

### Statistical analysis

Means and standard deviations were calculated for all variables at baseline, post-test and follow-up. To test whether baseline, post-test and follow-up were equal, we performed an analysis of variance (ANOVA) with repeated measurements. Comparisons between the average scores at baseline, post-test and follow-up on the three functional performance tests (according to the stopwatch time) and daily physical activity (both sensor- and self-reported outcomes) were performed using a paired sample t-test for normally distributed variables. As the scores on the functional performance tests were skewed, a gamma distribution was used. Bonferroni adjustments for multiple comparisons were performed to assess the periods responsible for significant outcomes. Cohen’s *d* was calculated for baseline versus post-test measures, post-test versus follow-up measures and baseline versus follow-up measures using the following formula: Cohen’s *d* = (M_1_ – M_2_) / SD_pooled_, where M is the mean and SD the standard deviation. To interpret the effect of Cohen’s *d*, the following benchmarks were used: 0.01–0.20 for a very small effect, 0.20–0.50 for a small effect, 0.50–0.80 for a medium effect and > 0.80 for a large effect [32, 33]. The maximum number of available data at each time point was used. No data imputation was applied for missing data. Because of the small data set, winsorizing was applied for outliers after data inspection. SPSS statistical software (version 24.0, IBM SPSS, Chicago) was used.

## Results

### Participants

Forty pre-frail (mean GFI score 4.4 ± 0.5) participants were included, 15 male and 25 female. Their mean age was 81.3. ± 4.7 years. Twenty-five participants had previous experience with a PC or tablet PC. One participant owned a smartphone. Characteristics of the participants are summarised in Table 1.

**Table 1:** Characteristics of participants

	Participants (***N*** = 40)
**Gender (n) (m/f)**	15/25
**Age (years)**	81.3 ± 4.7
**GFI (score)**	4.4 ± 0.5
**FES-I**	9.8 ± 2.8
**BMI (kg/m**^**2**^**)**	27.9 ± 4.1^a^
**SQUASH**	1243.5 ± 766.4
**PA (%) TOL**	66.0 ± 26.2^b^
**TUG (s)**	13.7 ± 5.8^a^
**STS (s)**	1.4 ± 0.5^a^
**CR (s)**	19.9 ± 7.9^a^
**Computer experience (n) (y/n)**	25/15
**Smartphone owner (n) (y/n)**	1/39
**Internet type (n) (3G/4G/Wi-Fi)**	17/11/12

^a^Two missing values/ ^b^Eight missing values

Values are mean ± standard deviation unless otherwise specified

*n* Number, *m/f* Male/female, *GFI* Groningen Frailty Indicator, *FES-I* Falls Efficacy Scale International, *BMI* Body mass index, *kg/m* kilogram/metre, *s* seconds, *y/n* yes/no, *PA* Physical activity, *TOL* Time On Legs, *TUG* Timed Up & Go test, *STS* Sit-to-stand test, *CR* Chair-Rise test

Twenty-one participants completed the entire programme. The average progression through the 18 levels was 7.9 within the first 3 months of intervention and 3.8 levels within the second three months. Three participants reached a plateau during the last 3 months, all others kept increasing their level of training during the intervention.

### Functional performance

All but two participants were able to perform the required repetitions of STS, TUG and CR tests at all three measurement appointments. One participant was unable to perform any physical test at follow-up due to an amputated toe. All but four participants were able to perform their CR tests twice at all appointments. Those four participants only performed one CR test at any given appointment. Results obtained by using the stopwatch were used for the analyses. Table 2 summarises the STS, TUG and CR average scores of the participants at all measurement points.

**Table 2:** Mean scores on functional performance tests at all measurement points

	Baseline (s)	Post-test at 3 months (s)	Follow-up at 6 months (s)	P Baseline vs. Post-test	P Baseline vs. Follow-up	P Post-test vs. Follow-up	Cohen’ ***d*** Baseline vs. Post-test	Cohen’ ***d*** Baseline vs. Follow-up	Cohen’***d*** Post-test vs. Follow-up
**STS (*N*** **= 38–23-20)**	1.4 (1.19–1.87)	1.3 (1.05–1.45)	1.2 (1.03–1.28)	0.200	0.137	0.820	0.20	0.49	0.24
**TUG (*N*** **= 38–23-20)**	13.4 (10.91–13.14)	11.0 (9.48–12.18)	10.5 (9.50–11.89)	0.006*	0.002*	0.611	0.66	0.81	0.21
**CR (*N*** **= 38–23-20)**	19.5 (15.40–27.09)	17.3 (15.46–20.17)	17.5 (13.94–20.50)	0.032	0.053	0.917	0.42	0.34	0.04

Values are means (95% confidence interval)

*STS* Sit-to-stand test, *TUG* Timed Up & Go test, *CR* Chair-Rise test, *N* baseline-post-test-follow-up, *s* seconds

STS: F (2.78) = 1.408 (*p* = 0.251); TUG: F (2,78) = 6.522 (*p* = 0.002); CR: F (2.78) = 3.018 (*p* = 0.055)

*Significant difference (*P* ≤ 0.016 after Bonferroni correction)

Average time needed to perform the STS decreased from baseline to post-test as well as during the unsupervised period, although these changes were not significant (*p* = 0.200, *p* = 0.137 and *p* = 0.820). Average time needed to perform the TUG showed a significant decrease from baseline to post-test (*p* = 0.006) and from baseline to follow-up (*p* = 0.002). Although not from post-test to follow-up (*p* = 0.611). Average time needed to perform the CR decreased from baseline to post-test (*p* = 0.032) and increased from post-test to follow-up (*p* = 0.917), although both non-significantly. The decrease from baseline to follow-up was also non-significant (*p* = 0.053). The largest decrease occurred during the supervised period.

Absolute improvements in all three functional tests varied between 0.1 and 2.9 s. One very small effect, six small effects, one medium effect and one large effect were found.

### Daily physical activity

There was a non-significant increase of self-reported physical activity during the trial (*p* = 0.584 and *p* = 0.833), with the highest rise in the supervised period (*p* = 0.732). Overall there was a non-significant increase in objectively measured daily physical activity during the supervised period (*p* = 0.881), with a drop-back below baseline level at follow-up (*p* = 0.362 and *p* = 0.309). Changes in objectively measured physical activity varied between − 5 and 2% and in self-reported physical activity between 97.1 and 298.0 min per week and can be considered (very) small effects. Table 3 summarises the daily physical activity scores of the participants at all measurement points.

**Table 3:** Mean scores on daily physical activity at all measurement points

	Baseline	Post-test at 3 months	Follow-up at 6 months	P Baseline vs. Post-test	P Baseline vs. Follow-up	P Post-test vs. Follow-up	Cohen’***d*** Baseline vs. Post-test	Cohen’***d*** Baseline vs. Follow-up	Cohen’***d*** Post-test vs. Follow-up
**SQUASH minutes/week (*N*** **= 40–23-20)**	1243.5 (1140.0–1906.7	1444.4 (1109.5–1995.1)	1541.5 (1176.5–2014.1)	0.732	0.584	0.833	0.25	0.38	0.12
**Physical activity (%)** **(*N*** **= 32–21-16)**	66.0 (57.70–85.66)	68.0 (48.52–80.54)	63.0 (50.48–73.80)	0.881	0.362	0.309	0.07	0.13	0.20

Values are means (95% confidence interval)

*N* baseline-post-test-follow-up

Physical Activity: F (2.47) = 0.160 (*p* = 0.853); Squash: F (2.37) = 0.602 (*p* = 0.553)

*Significant difference (*P* ≤ 0.01625 after Bonferroni correction)

## Discussion

In this pilot study an initial insight into the effectiveness of a home-based exercise programme for pre-frail older adults with independent use of novel technology was gained. Training was performed under remote supervision during the first 3 months of the trial, after which participants trained without supervision for 3 months. Improvements varying between (very) small and large effects were seen in functional performance, of which the change in TUG was statistically significant. Changes in daily physical activity had (very) small, non-significant effects. Overall, improvements were mainly observed during the supervised period. This result can be interpreted in different ways. First of all it could be that participants already reached a high level during the supervised period leaving less room for improvement during the unsupervised period. However to our opinion a more plausible explanation is that although pre-frail older adults are able to use this novel technology independently additional regular remote supervision probably is necessary to preserve effectiveness in the home-based exercise programme.

In terms of functional performance, TUG showed significant improvement with small-to-large effects, particularly during the supervised period. The improvement seen in STS and CR were not significant and could therefore be regarded as a ‘non-change’ or maintenance, although these changes showed (very) small effects. The significant improvement in TUG reflects the results obtained by Maillot et al., who found a significant decrease in TUG time in their intervention group receiving WiiMote and Balance board training at home for 12 weeks [34]. Cicek et al. found that by administering a WiiFit and WiiBalance training during 8 weeks TUG performance improved significantly more in the intervention group than in the physical activity and control groups [35]. Concerning functional performance, the results of this programme are comparable to those summarised in a systematic review by Laufer et al., reporting significant and nonsignificant positive but very variable effects of home-based programmes integrating Wii technology [36]. In conclusion, the improvements for functional performance seen in our intervention follow the results of similar interventions from the literature, although our results showed (very) small to large effects and only TUG showed significant improvement.

As the sensor was a research prototype; self-reported physical activity as measured with the SQUASH was the primary outcome regarding daily physical activity. Self-reported physical activity showed an improvement, although these changes had a (very) small effect and were non-significant. In addition it must be kept in mind that this outcome may be the result of an overestimation by the SQUASH, as people tend to overestimate their physical activity level [33]. Objectively assessed daily physical activity showed an improvement during the supervised period and a deterioration during the unsupervised period. These results should be regarded as maintenance of physical activity, as they were also not significant and had very small effects. A stronger positive effect in physical activity is found in literature. For example, Morey et al. reported a significantly greater change in weekly durations of strength and endurance training after 12 months of training with infrequent telephone coaching [37]. The difference in results is possibly due to the shorter intervention and smaller sample size of our study.

The improvements seen in functional performance were larger and stronger than those seen in daily physical activity. Apparently, improvement of functional performance does not necessarily result in the same improvement in physical activity behaviour. This difference may be attributed to coaching. Stimulating progression in exercises across levels was done in consultation with the coach using motivational feedback. Improvement of daily physical activity was only encouraged by a visual graph showing the progression. Feedback and education seem therefore important towards improvement or behavioural change, as described in literature [38].

The results of this pilot must be interpreted within the context of the development of novel technology for older people. As a result this study had several strengths but also limitations. First of all older people are not the easiest group to target. As stated in the design paper, the initial goal was to include 50 participants [24]. This was difficult to realise because candidates did not always meet the defined inclusion criterion of pre-frailty, as indicated by the GFI score. In addition, while all candidates were pre-frail according to the GFI, the majority of their baseline outcome measures on the STS, TUG, CR and physical activity assessments indicated they were more physically active and less physically frail than originally expected [14, 17, 18, 29, 30]. An explanation can be found in the nature of the instrument used for inclusion. The GFI measures not only physical aspects but psychosocial aspects too [25], hence subjects could be classified as pre-frail yet still functioning at an acceptable level physically.

Secondly, the drop-out rate during the trial was higher than expected (48% instead of 20%). In total, nineteen participants did not complete the study. For the statistical analyses the maximum number of available data at each time point was used. An additional sensitivity analyses comparing completers with non-completers did not result in different outcomes. During the initial phase of the study major internet connectivity problems were encountered, as a result of which eight participants dropped out. Additionally, seven stopped for medical reasons not related to the exercise programme, three because of illness of their spouse, and one died. These reasons can be considered inherent to the age of our target group. Notably, at baseline dropouts had a significantly higher FES-I score (*p* = 0.03), less computer experience (*p* < 0.01), lower self-reported physical activity ((*p* < 0.01) and less advanced internet (*p* < 0.01) compared to completers. On the other hand, in a previous paper on the feasibility of this home-based exercise programme it was concluded that adherence of completers was considered sufficient during the 3 months of supervision (75.8%) and in the unsupervised period (62.4%). The feasibility of the programme was rated by completers with a 4.3 (after 3 months) and 4.2 (after 6 months) on a scale of 1 to 5 (higher meaning more positive) [39]. Still, the overall high drop-out rate can be considered a major limitation of the power of this intervention, and as a result the intended subgroup analyses were not conducted [24]. Imputation of missing data was not considered to be of added value due to the small sample size.

Thirdly, stopwatch data were used to measure time needed for the performance tests instead of data from the necklace-worn sensor, as these latest data were not considered reliable enough due to measurement errors. A final limitation of the study is rooted in its design as a pilot trial without a control group. This design can only provide a first indication of its effectiveness, but since there is no control group the effectiveness cannot be definitely stated. However, the use of pilot studies to gather information on the potential effectiveness and feasibility of a technology before progressing to a larger randomised controlled trial is common in this field, where large costs are encountered when developing and testing new technology.

This study also had several strengths. In the first place, for both functional performance and daily physical activity mainly objective measures were used. Secondly, the three-month supervised period and the three-month non-supervised period together provide a time span that should suffice to pinpoint the long-term effects [33]. Lastly, the intervention was well-tailored to participants’ preferences for exercise burden.

## Conclusion

Home-based exercising using novel technology seems promising for functional performance and physical activity enhancement. However the results of this pilot should be further consolidated in a comparative trial with a larger sample size. Lessons learned which can be incorporated in this future trial are that remote supervision seems to be important for the effectiveness of a home-based exercise programme using a tablet application. In addition, future research should preferably incorporate a more in-depth analysis of the effect of integrating regular remote contact with a coach as opposed to motivational strategies delivered by tablet technology alone. Aspects to be integrated into this evaluation should be frequency, specific content and type of contact. Furthermore, a stable internet connection enabling reliable insight into sensor-wearing adherence as well as exercise and physical activity behaviour are key to adequate performance of these interventions. The feedback on exercise and daily physical activity behaviour should be available to both coach and participant on a daily basis. Feedback on exercise behaviour should preferably control for actual performance of the exercises based upon sensor data instead of playing the videos.

Overall it can be concluded that this pilot study showed positive results varying between very small and large effects in levels and maintenance of functional performance and daily physical activity. These results were more prominent during the supervised period of the intervention, so weekly contact with a coach seemed important. As the power of this pilot trial is limited, the effectiveness of the intervention needs to be further established in a randomised controlled trial.

## Supplementary Information

**Additional file 1.** Table exercise program contents.

## Data Availability

The datasets used and/or analysed for the current study are available from the corresponding author upon reasonable request.
