# Supplementary material for: Effectiveness of an individually tailored home-based exercise rogramme for pre-frail older adults, driven by a tablet application and mobility monitoring: a pilot study
Source: Eur Rev Aging Phys Act. 2021 Jun 21;18:10. doi: 10.1186/s11556-021-00264-y (PMC8215778; doi:10.1186/s11556-021-00264-y)
Supplement: Supplementary file 1 — Additional file 1. Table exercise program contents. [file 11556_2021_264_MOESM1_ESM.pdf]

Additional file 1 Table exercise program contents

Level<br>Exercise	Target	1	2	3	4	5	6	7	8	9	10	11	12	13	14	15	16	17	18
**Standing-eyes closed (seconds)**	**Balance**	10	10	10	20	20	20	30	30	30	30	30 (10 US)	30 (10 US)	30 (10 US)	30 (20 US)	30 (20 US)	30 (US)	30 (US)	30 (US)
**Back extension**	**Balance**	1	2	2	3	3	3	5	5	5	5	5	8	8	8	8	10	10	10
**Standing; leg front-back**	**Balance**	5L<br>5R	5L<br>5R	8L<br>8R	8L<br>8R	10L<br>10R	10L<br>10R	10L<br>10R	10L<br>10R	10L<br>10R	10L<br>10R	15L<br>15R	15L<br>15R	15L<br>15R	15L<br>15R	20L<br>20R	20L<br>20R	25L<br>25R	25L<br>25R
**Turn trunk left-right**	**Balance**	3L<br>3R	4L<br>4R	5L<br>5R	5L<br>5R	8L<br>8R	10L<br>10R	10L<br>10R	16L<br>16R	16L<br>16R	16L<br>16R	16L<br>16R	20L<br>20R	20L<br>20R	20L<br>20R	20L<br>20R	30L<br>30R	30L<br>30R	30L<br>30R
**Knee bends**	**Balance**	1	2	2	3	3	3	5	5	8	5 (1 US)	5 (1 US)	8 (1 US)	8 (1 US)	8 (2 US)	8 (2 US)	10 (2 US)	10 (3 US)	10 (3 US)
**5 steps sideways**	**Balance**	1 stp L; 1 stp R	2 stp L; 2 stp R	3 stp L; 3 stp R	3 stp L; 3 stp R	4 stp L; 4 stp R	1	1	2	2	2	2	3	3	4	4	5	5	5
**Tandem stance (seconds)**	**Balance**	20	20	20	30	30	30	40	40	50	50	50	60	60	60	60	60	60	60
**Knee extensor**	**Strength**	8L<br>8R	10L<br>10R	10L<br>10R	16L<br>16R	20L<br>20R	20L<br>20R	20L<br>20R	20L<br>20R	20l<br>20R	20L<br>30R	30L<br>30R	30L<br>30R	20L<br>20R<br>*hold*	20L<br>20R<br>*hold*	20L<br>20R<br>*hold*	30L<br>30R<br>*hold*	30L<br>30R<br>*hold*	30L<br>30R<br>*hold*
**Knee flexor**	**Strength**	8L<br>8R	10L<br>10R	16L<br>16R	16L<br>16R	20L<br>20R	20L<br>20R	20L<br>20R	20L<br>20R	20L<br>20R	30L<br>30R	30L<br>30R	30L<br>30R	20L<br>20R<br>*hold*	20L<br>20R<br>*hold*	30L<br>30R<br>*hold*	30L<br>30R<br>*hold*	30L<br>30R<br>*hold*	30L<br>30R<br>*hold*
**Hip abductor**	**Strength**	5L<br>5R	5L<br>5R	8L<br>8R	8L<br>8R	10L<br>10R	10L<br>10R	10L<br>10R	10L<br>10R	10L<br>10R	10L<br>10R	15L<br>15R	15L<br>15R	15L<br>15R	15L<br>15R	20L<br>20R	20L<br>20R	20L<br>20R	25L<br>25R

**One leg stand (seconds)**	**Balance**	10	10	10	20	20	20	30	30	30	30	30 (10 US)	30 (10 US)	30 (10 US)	30 (20 US)	30 (20 US)	30 US	30 US	30 US
**Heel stand (hs)** *Level 8>: heel walk*	**Balance**	8	10	10	16	16	16	20	1	3	5	8	8	10	10	16	16	20	24
**Toe stand (ts)** *Level 8>: toe walk*	**Balance**	8	10	10	16	16	20	20	1	3	5	8	8	8	10	16	16	20	24
**TUG**	**Balance**	1N	1N	1N	2N	2N	2N	3N	3N	3N	3N 1F	3N 1F	3N 1F	3N 2F	3N 2F	3N 3F	3N 3F	5N 3F	5N 3F
**CR**	**Balance**	1	1	1 NH	1 NH	1 NH	2	2	2 NH	2 NH	2 NH	3	3	3	3 NH	3 NH	4	4	4 NH
**Level video duration (minutes)**		10	12	14	17	18	19	20	22	21	24	26	28	26	27	32	37	39	40

US = unsupported (optional)
L = Left leg or to the left
R = Right leg or to the right

N = Normal speed
F = Fast speed
NH = No hands

Added ankle weights of 1 kilogram to the exercise to increase burden
